# In Vitro Studies of Genistein Lipophilic Derivatives as Potential UV Radiation Protectors

**DOI:** 10.3390/ph17091166

**Published:** 2024-09-03

**Authors:** Magdalena Skonieczna, Kinga Plasa, Ewa Borowska, Agata Jakubowska, Wiesław Szeja, Anna Kasprzycka

**Affiliations:** 1 Department of Systems Engineering and Biology, Faculty of Automatic Control, Electronics and Computer Science, Silesian University of Technology, Akademicka 16, 44-100 Gliwice, Poland; magdalena.skonieczna@polsl.pl; 2 Biotechnology Centre, Silesian University of Technology, Krzywoustego 8, 44-100 Gliwice, Poland; kinga.plasa@polsl.pl (K.P.); ewa_borowska@onet.eu (E.B.); 1agata.jakubowska@gmail.com (A.J.); 3 Department of Organic, Bioorganic Chemistry and Biotechnology, Faculty of Chemistry, Silesian University of Technology, Krzywoustego 4, 44-100 Gliwice, Poland; wieslaw.szeja@adres.pl

**Keywords:** genistein, genistein derivatives, ROS, antioxidant properties, UV radiation

## Abstract

The major environmental factor responsible for skin cancer is ultraviolet (UV) radiation, present in sunlight. UV radiation is directly linked to the production of reactive oxygen species (ROS), which accumulate in exposed cells and cause serious damage. The antioxidant systems present in cells cannot always sufficiently neutralize the ROS. Therefore, supplementation with exogenous antioxidants has been proposed. The antioxidant properties of some isoflavones, such as genistein, have already been well-proven. Genistein has limited bioavailability. However, its derivatives, with increased lipophilicity, could facilitate its transfer into cells, where they can expose its antioxidative potential. This study aims to investigate three genistein derivatives, with greater lipophilicity than the native compound, regarding their cytotoxicity, antioxidative properties, and effect on the cell cycle in normal human dermal fibroblasts (NHDF) and a melanoma cancer cell line (Me45). Results showed that lipophilic modification of the genistein molecule changes the biological response of NHDF and Me45 cell lines to UV-C radiation, but the lipophilicity cannot be directly linked with the activity of the compounds. A comparison of the effects of the genistein derivatives on healthy and cancerous cells suggests that their mode of action strongly depends on the type of cell involved.

## 1. Introduction

It is believed that the major environmental factor responsible for skin cancers, especially malignant melanoma, and early skin aging is the ultraviolet (UV) radiation present in sunlight [1]. Short-term contact of the skin with UV radiation causes a suntan, while long-term exposure can lead to serious skin damage, including carcinogenesis and accelerated skin aging [2]. UV radiation is directly linked to the production of reactive oxygen species (ROS), which accumulate in exposed cells causing the peroxidation of membrane lipids and the formation of hydrogen peroxide and secondary products such as aldehydes, and ultimately leads to DNA damage [3].

Antioxidants in the cell membrane and cytoplasm of cells can prevent damage and neutralize ROS [4]. Intracellular antioxidant systems can be supported by supplementation with exogenous antioxidants of natural or synthetic origin. Antioxidants can be compounds of enzymatic and non-enzymatic origin. Endogenous antioxidants include enzymes, low molecular weight compounds, and cofactors. Among the non-enzymatic antioxidants, many are present in food. Such antioxidants belong to different classes of compounds, but polyphenols are the largest group [5,6]. Polyphenols include phenolic acids and flavonoids. Other dietary antioxidants include vitamins, carotenoids, and minerals [6,7,8].

It is estimated that two-thirds of human cancers are associated with multiple gene mutations, which could be prevented by enriching the diet with antioxidants from the polyphenol group [7,9,10]. The bioavailability of dietary antioxidants depends on several factors. The main disadvantages of food-derived antioxidants are their poor solubility, poor penetration of cell membranes, instability, and breakdown in the gastrointestinal tract [7,11]. Consequently, the oral intake of antioxidants may not be effective due to their poor biopharmaceutical properties [7]. To significantly improve their efficacy, the mode of ingestion needs to be altered or chemically modified so that the derivatives have better physical, chemical, biopharmaceutical, and pharmacokinetic properties [7,11]. For many years, antioxidants were obtained from natural sources, mainly plants. Due to the increased chemical demand, synthetically derived antioxidants have become available [12]. These compounds, unlike drugs, act non-specifically and are pleiotropic [13]. Flavonoids are compounds belonging to the group of polyphenols naturally found in plants, mainly in leaves, seeds, bark, and flowers, and protect them from UV radiation, pathogens, and herbivores. To date, more than 8000 flavonoids have been identified [14,15]. Anthocyanin pigments present in flowers attract pollinating insects and are responsible for the characteristic red and blue colors of berries, wine, and some vegetables [16,17,18]. The physical, chemical, biological, and metabolic properties of these compounds depend on the type and location of the substituents present in the basic polyphenol structure. For antioxidant properties, the degree of hydroxylation is most important [19,20,21].

Flavonoids are found in many plant species. As a result, flavonoids are a common component of human foods. They are absorbed from the gastrointestinal tract and excreted unchanged or as metabolites present in the urine and feces [22,23]. Flavonoids are believed to be non-toxic. They have a broad spectrum of biological properties, including anti-bacterial, anti-viral, anti-inflammatory, and anti-allergic [22,24,25]. They also influence vasodilation and inhibit lipid peroxidation and thrombocyte aggregation. Once absorbed, they can prevent the cytotoxicity associated with free radical formation and lipid peroxidation, which, in turn, are associated with the aging process and numerous chronic diseases [26,27]. Epidemiological studies have shown an inverse relationship between flavonoid intake and coronary heart disease mortality. This is explained by the inhibition of low-density lipoprotein (LDL) oxidation and reduced thrombocyte aggregation [28,29,30].

**Figure 1:**
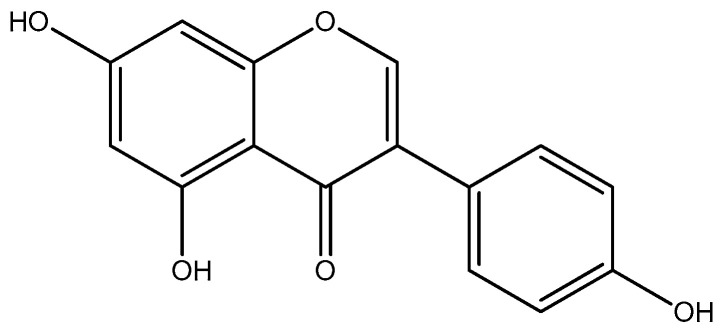
Chemical structure of genistein.

One flavonoid with antioxidant properties is genistein (Figure 1). Genistein has a high propensity to remove free radicals, so its antioxidant potential is used in various therapies [31,32].

Genistein is a potent anti-cancer agent (both in terms of prevention and treatment), and the considerable interest in it has led to the discovery of its many biological activities [33,34,35]. Despite its relatively simple chemical structure, genistein is involved in many processes in the body. Genistein is a phytoestrogen which has different affinities for the estrogen receptors ERα and ERβ, binding 20–30 times more strongly to ERβ than ERα [36]. Although some of the biological activity of genistein is attributed to its competitive affinity for estrogen receptors, it may also modify cancer risk. At the cellular level, genistein’s activity includes the inhibition of tyrosine kinases, resulting in the inhibition of phosphorylation of the proteins required for cell division [34,35,37]. Genistein also affects topoisomerase II, preventing DNA repair and replication [35,38]. In addition, genistein inhibits the autophosphorylation of epidermal growth factor (EGF), which is overexpressed in tumors. Interestingly, genistein can promote (by differentiating tumor cells) or inhibit (by inducing apoptosis) mutagenesis and can cause or counteract oxidative DNA damage [39,40]. These effects have been observed over a wide range of concentrations (1 μM–0.5 mM) [40]. All these properties make genistein a potential anti-cancer drug. Beneficial effects have also been observed in osteoporosis, cardiovascular disorders, and menopause. Genistein’s greatest strengths as an effective drug are its pleiotropic effects in cells and its low toxicity [34,35,41].

Due to its limited bioavailability, research is currently being conducted to improve genistein’s physical, chemical, and biological properties by modifying its primary structure. These modifications also aim to preserve its antioxidant properties in normal, healthy cells, while maintaining its antiproliferative properties against tumor cells [42]. Increasing the bioavailability of genistein by isolating its lipophilic derivatives greatly facilitates its transport into cells, which is particularly important in the treatment of psoriasis or vitiligo. Treatment of these conditions with irradiation has undesirable side effects, including the generation of ROS in the cells exposed to radiation. The introduction of formulations containing antioxidants means that therapies involving UV radiation are less severe in terms of side effects, especially for the healthy cells undergoing radiation. Therefore, oxidative stress, which ultimately leads to the damage of the cell’s genetic material (DNA and RNA) [39] is minimized. Accumulated damage due to radiation, mainly DNA strand breaks, leads to mutations either through abnormal pairing of modified bases during DNA replication or due to excision repair errors [43]. Failure of the repair systems can ultimately lead to apoptotic cell death [44]. The use of a system which eliminated both ROS and cancer cells using selectively acting genistein derivatives was found to significantly reduce this problem.

Within the scope of preliminary studies, we investigated the protective effect of genistein carbonates (n-pentyl-7-O genistein carbonate (W5-Gen) and benzyl-7-O-genistein (WBn-Gen)), and the genistein 4′-laurate ester in cultured normal human dermal fibroblasts (NHDFs) and human melanoma Me45 cancer cells that had been subjected to UV-C irradiation. The tested derivatives differ in the length of the R substituent and, therefore, lipophilicity. The more carbon atoms present in the substituent, the greater the lipophilic properties. This experimental model corresponded to that of human skin exposed to the negative effects of sunlight. The results obtained provide preliminary answers regarding whether the genistein derivatives could have potential applications in the treatment and prevention of sunburn, or in cosmetology as an element of photoprotective formulas which delay neoplastic transformation. Additionally, whether genistein analogs fulfill their antiproliferative function toward melanoma cells will be studied.

The present study aimed to test the ability of genistein and its derivatives to improve the protection of human cells against UV-C irradiation. Moreover, we wanted to test whether adding lipophilic substituents to the basal genistein molecule would improve its biological biocompatibility and its efficiency in ROS scavenging. All tests were performed on both healthy and cancerous cell lines in parallel to compare the effects. The first step was to evaluate the half-maximal effective concentration (EC_50_) values for all the compounds to select the concentrations for further tests. Samples in all the subsequent tests were divided into two groups: not irradiated or irradiated with UV-C, which served as an inducer of abundant ROS production in cells. We tested the cytotoxicity of the compounds and measured the ROS levels to evaluate whether the compounds protected the cells from oxidative stress. Moreover, the genotoxic effects were evaluated using a micronucleus assay and by investigating the cell cycle progression. The present study provides an overview of the genistein derivatives’ effects on healthy and cancerous cell lines and verifies their radiation-protective and antioxidant properties.

## 2. Results and Discussion

### 2.1. Determination of EC_50_ Values—MTS Assay

The investigated compounds were evaluated using various tests. First, cytotoxicity potential was studied by monitoring and determining the effect of the compound dosage non cells. The EC_50_ values were calculated using the MTS assay.

**Figure 2:**
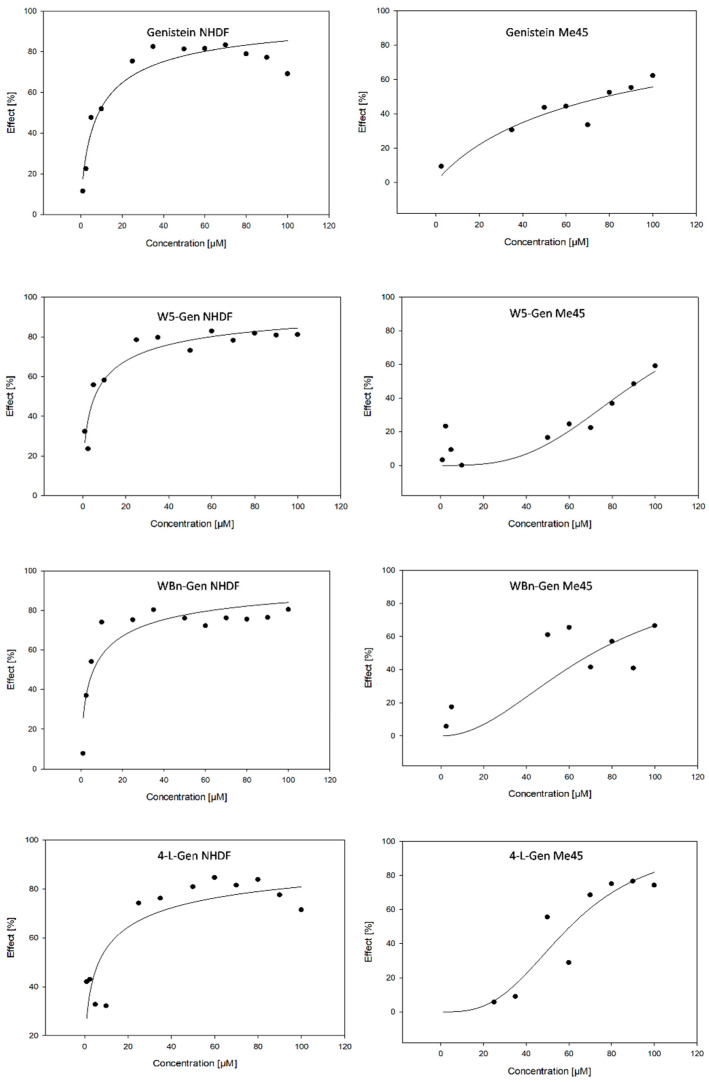
Effect–dose curves of genistein and its derivatives on NHDFs and Me45 cells.

The cytotoxicity of both cell lines appeared to be dose-dependent (Figure 2). The EC_50_ values of genistein and its derivatives (i.e., W5-Gen, WBn-Gen, and 4′-L-Gen) for the NHDF and Me45 cell lines are presented in Table 1. The results showed that the investigated compounds are much more toxic toward normal fibroblasts (8.6 µM EC_50,Gen_ for NHDFs) compared to cancer cells (78.2 µM EC_50,Gen_ for Me45 cells). In the case of NHDFs, the genistein derivative EC_50_ values were lower than the EC_50_ value of Gen, which indicates that the modification of genistein’s structure increases its toxicity towards healthy fibroblasts. The least toxic compound towards NHDFs was genistein, which is consistent with previous studies [45]. Moreover, the higher the lipophilicity, the more toxic the compound.

The response of cancer cells to incubation with the investigated compounds was different. The most toxic derivative for NHDFs (i.e., W5-Gen) exhibited the lowest cytotoxicity towards Me45 cells (EC_50_ = 92.2 µM). Similar to NHDFs, the derivatives WBn-Gen and 4′-L-Gen affected the survival of the Me45 cells more than genistein itself; the calculated EC_50_ values being 71.2 µM and 60.5 µM, respectively. The comparison of the EC_50_, especially for W5-Gen, suggests that the toxic effect on these two cell types probably occurs via distinct mechanisms.

**Figure 3:**
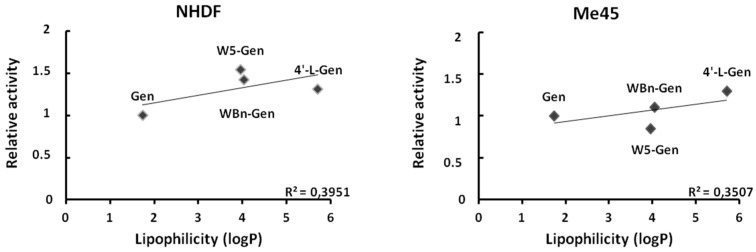
Dependence of the relative activities of the compounds versus their lipophilicity.

Next, we assessed the correlation between the cytotoxicity and lipophilicity of the investigated compounds. Figure 3 represents the dependence of the relative activity (EC_50,Gen_/EC_50,derivative_) on lipophilicity (log P). The low R^2^ values of the plots (0.3951 and 0.3507 for NHDFs and Me45 cells, respectively) indicate that there is no linear dependence between these two parameters. Therefore, it is likely that the structure of the lipophilic part of the compound affects the activity of the compound, but not the lipophilicity itself.

### 2.2. Protective Potential against UV Radiation—MTS Assay

**Figure 4:**
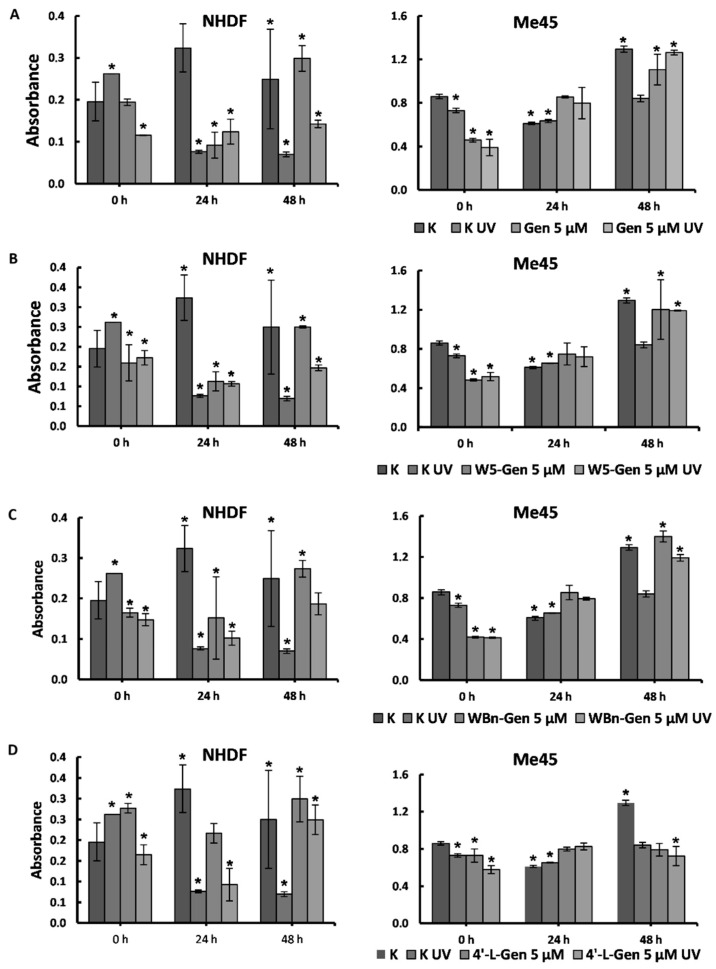
Survival of NHDFs and Me45 cells treated with genistein (**A**) and its derivatives (**B**–**D**) and exposed to UV radiation, as estimated using the MTS assay at different time points. Results are presented as mean ±SD, calculated from three experiments and compared to the untreated controls (K) at the 0 h time point. Statistical significance was calculated using the *t*-test where *p* < 0.05 and is indicated by an asterisk (*).

To limit the proliferation of cancer cells, drugs should be administered at a concentration exceeding the values of EC_50_. However, these drug compounds are extremely toxic to healthy cells. Considering the fact that the compounds investigated in this study were intended to serve as diet supplements rather than cancer drugs, we performed all tests using a concentration of 5 μM. This concentration does not significantly decrease the survival of Me45 cells, and thus 5 μM was used to verify the biological activity of the selected isoflavones in healthy and cancerous cells exposed and not exposed to UV radiation. These experiments aimed to estimate the metabolic effects of selected compounds on healthy and cancerous cells shortly after treatment with genistein or its derivatives, following up 24 h and 48 h after treatment to observe the dynamics of cell viability. The results are presented in Figure 4. At each time point, the absorbance of the control cells (K) and the absorbance of the control cells exposed to UV radiation (K UV) were compared to the absorbance of the cells treated with isoflavones (Gen/W5-Gen/WBn-Gen/4′-L-Gen) and to the absorbance of the cells treated with isoflavones and UV radiation exposure (Gen/W5-Gen/WBn-Gen/4′-L-Gen UV).

The highest viability of cells was observed in the control samples exposed to UV (K UV) for NHDFs. UV radiation exposure likely causes a short-term increase in the cells’ ability to minimize the negative effects of oxidative stress.

The addition of genistein and its derivatives does not change the viability of the cells after 1 h of incubation with NHDFs. Additionally, after 1 h of incubation, the observed absorbance was always much lower in the treated samples than in the control UV-treated samples and thus did not reveal a protective effect in the irradiated samples. Most likely, chemical and physical stressors triggered the cells’ oxidative stress, which increased the mortality.

After 24 h of incubation, the viability of the control samples increased significantly, whereas the viability of all treated samples, both irradiated and not irradiated, decreased in comparison to the 1 h incubation. Therefore, after 24 h, isoflavones exhibited a negative effect on NHDFs. Improved viability was observed after 48 h of treatment, especially for K UV samples and genistein-treated samples exposed to UV. In all cases, the viability of the cells treated with the compounds was higher than the viability of the samples exposed only to UV radiation. Although the investigated compounds negatively affect healthy cells, they act as a protective agent against UV radiation. No significant changes in Me45 cell viability were observed. The incubation of Me45 cells with the investigated compounds resulted in reduced cell viability. The strongest effect was observed in cells treated with genistein (50% mortality compared to control untreated cells), whereas treatment with 4′-L-Gen did not affect viability dramatically (~10% mortality). After 24 h of incubation, the viability of the cancer cells exceeded the viability of both K and K UV cells, and no differences between the genistein compound-treated and UV-exposed genistein compound-treated samples were observed. Therefore, isoflavones have a beneficial effect on the metabolism of cancer cells.

After 48 h, this beneficial effect was evident for Me45 cells treated with Gen, W5-Gen, and WBn-Gen. In the case of the 4′-L-Gen treatment, the viability of the cells treated with the derivative, both exposed and not exposed to UV radiation, was much lower than in the control samples (~50%).

From these experiments, one can conclude that the investigated compounds exhibit a much stronger protective effect towards cancer cells. However, they can also protect healthy cells from the negative effects of UV radiation, especially 48 h after UV exposure. The survival fraction of cell viability in comparison to the untreated controls is presented in Figure S4, Supplementary Materials.

### 2.3. Quantification of Intracellular ROS

The presence of a new agent, either physical (i.e., UV radiation) or chemical (i.e., a new substance in the medium), can cause oxidative stress, which is characterized by the formation of numerous ROS. The abundance of ROS leads to a highly harmful state, which damages the cell and can eventually lead to many pathologies and cell death. Although cells are equipped with natural antioxidative mechanisms, in many cases these are insufficient and exogenous supplementation is necessary. Therefore, we aimed to investigate the antioxidative potential of genistein derivatives by measuring the amount of ROS formed in the cells exposed and not exposed to UV radiation.

ROS levels were measured in the cells treated with the investigated compounds without UV exposure to assess whether the presence of the chemicals can generate ROS. Subsequently, the ROS levels in cells treated with genistein derivatives were compared with the ROS levels in cells treated with genistein derivatives and exposed to UV radiation, to verify if these chemicals can neutralize the ROS caused by UV radiation.

**Figure 5:**
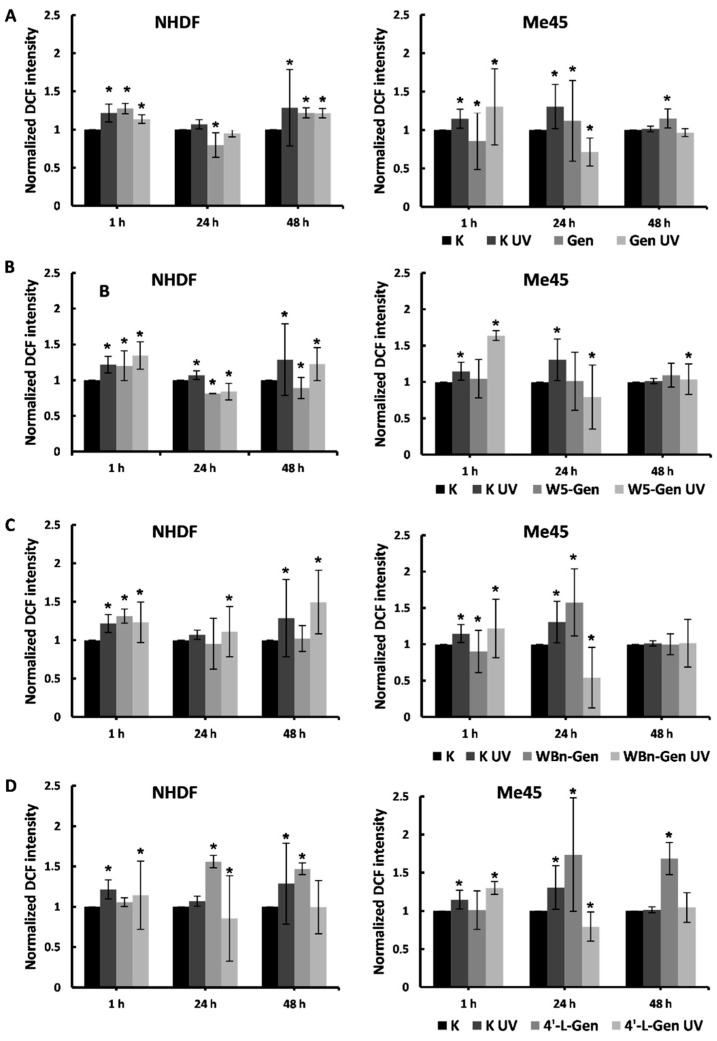
Changes in ROS levels in cells treated with (**A**) genistein (Gen), and its derivatives (**B**) W5-Gen, (**C**) WBn-Gen, and (**D**) 4′-L-Gen. Results are presented as mean ±SD calculated from three experiments and compared to the untreated controls (K) at the 1 h time point. Statistical significance was calculated using the *t*-test where *p* < 0.05 and is indicated by an asterisk (*).

Results are presented as normalized fluorescence, which is the value of the fluorescence emitted by each sample divided by the value of fluorescence emitted by the control sample (no exposure to genistein derivatives or UV radiation) (Figure 5).

The results did not prove that genistein and its derivatives could significantly reduce the amount of ROS in irradiated and non-irradiated NHDFs. Unfortunately, high error values prevented precise conclusions from being drawn.

Genistein and its derivatives increased natural ROS levels after the shortest incubation period at similar levels or even exceeding the ROS production of irradiated samples. A 1 h incubation period is likely too short for cells to recover from the stress caused by irradiation and the manual handling of the samples. However, after 24 h of incubation, the difference between ROS levels in K and K UV samples was not very significant. Furthermore, ROS levels in the cells treated with the tested compounds were lower than in the K and even the K UV samples. The cells treated with 4′-L-G were the exception, with increased ROS levels in the non-irradiated cells higher than after the irradiation treatment alone. On the other hand, this compound caused lower ROS levels in irradiated cells.

After 48 h of incubation, K and K UV samples greatly differed in the amount of ROS present compared to the previous time points. Mean ROS levels in Gen and Gen UV samples were comparable, similar to K UV-treated samples, and greater than the control K samples, which indicates no protective potential at this time point. Moreover, ROS levels in W5-Gen UV and WBn-Gen UV-treated samples were much higher than in cells treated only with the tested compounds and control K samples. Again, apart from the cells treated with 4′-L-G and irradiation, ROS levels were lower than in K UV.

The effects observed in Me45 cells were different. After 1 h of incubation, ROS levels in the cells treated with the tested compounds were similar to those of control K samples. There were more ROS in the cells treated with the compounds and irradiated than in the other samples. There was no protective effect of genistein derivatives observed. After 24 h of incubation, the ROS levels in the irradiated cells treated with the tested compounds were much lower than in K UV and even K samples. This effect was most significant between the samples treated with different compounds, indicating that these compounds can offer protection for cancer cells. After 48 h of incubation, the ROS levels between almost all sample types were similar, which also suggests a protective quality in the tested compounds.

### 2.4. Genotoxicity–Micronucleus (MN) Assay

**Figure 6:**
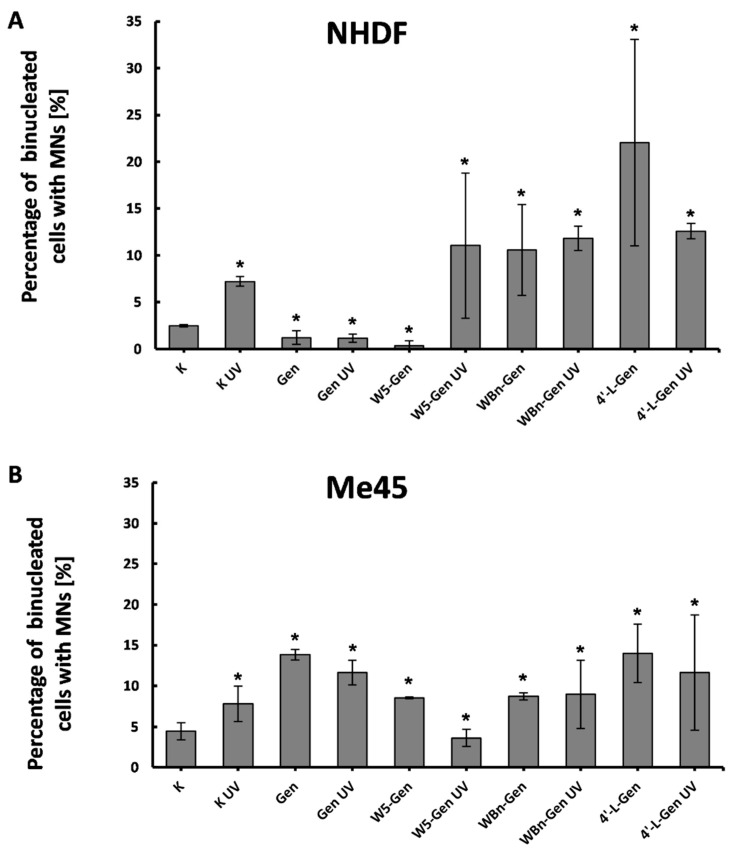
Percentage of binucleated cells with micronuclei (MN assay) in NHDFs (**A**) and Me45 (**B**) cells. Results are presented as mean ±SD calculated from three experiments and compared to the untreated controls (K). Statistical significance was calculated using the *t*-test where *p* < 0.05 and is indicated by an asterisk (*).

Measurement of ROS levels in the cells proved that genistein derivatives do not exhibit better antioxidant activity than genistein itself. The effect of genistein derivatives on genetic material was also studied to obtain a complete set of biological properties for these newly synthesized compounds. The genotoxicity of the investigated compounds was estimated via the micronucleus (MN) assay. Results are expressed as a percentage of the binucleated cells with MN compared to all binucleated cells. As predicted, the exposure of untreated cells to UV radiation results in damage to the genetic material (increased MN observed), both in control samples of NHDFs (Figure 6A) and in Me45 cells (Figure 6B).

The addition of genistein to the culture medium of NHDFs decreased the number of cells with MN compared to the control samples (K), suggesting that the compound can have a protective effect. The same protective effect was observed after irradiation. The presence of genistein derivatives in most of the cases increased the DNA damage in cells, and no protective effect regarding exposure to UV radiation was observed. However, in the case of W5-Gen, the number of cells not exposed to UV radiation with MN was much lower than in the K UV samples. Similarly, a synergistic destructive effect of the compounds and radiation combined was observed, with the number of cells with MN higher than in the cells exposed only to UV radiation. Similar levels of DNA damage (10.5–11%) were observed in WBn-Gen samples, both exposed and not exposed to UV radiation. In the case of 4′-L-Gen, the number of cells with MNs was much higher than in the K UV samples.

Comparing the number of MN formed in NHDFs and Me45 cells revealed that the Me45 cells are more susceptible to DNA damage than NHDFs. Even genistein increases the number of cells with MN, both in cells exposed and not exposed to UV radiation. Only the derivative W5-Gen shows a slight protective effect towards UV radiation-exposed cells. The rest of the investigated compounds cause genotoxic effects in both types of cells and increase the genetic instability of the cells. In the case of cancer cells, this can lead to an increase in virulence and metastasis.

**Figure 7:**
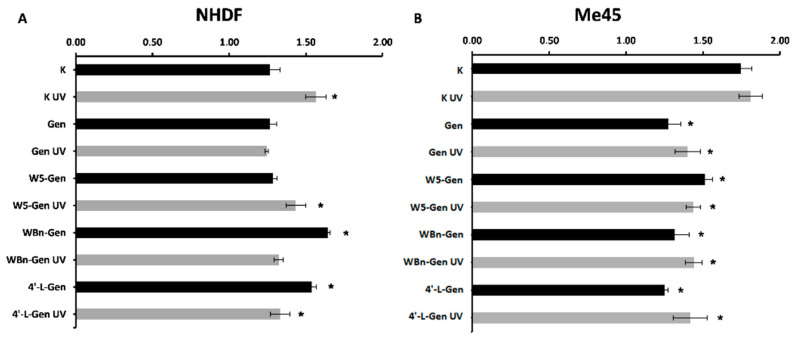
Nuclear division index (NDI) of NHDFs (**A**) and ME45 cells (**B**) treated with genistein and its derivatives, treated with or without UV radiation. Results are presented as mean ±SD calculated from three experiments and compared to the untreated controls (K). Statistical significance was calculated using the *t*-test where *p* < 0.05 and is indicated by an asterisk (*).

Additionally, the effect of the investigated compounds on cell division was evaluated using the nuclear division index (NDI) parameter. The values of NDI are presented in Figure 7. The NDI is higher in the K UV than in the K samples, which means that this physical agent enhances proliferation. Taking into consideration the number of cells containing MN, one can conclude that the increased ability of the cells to divide occurs in parallel with enhanced DNA damage.

Experiments performed on NHDFs demonstrated that the NDI values of genistein derivatives were comparable with the NDI values of control samples treated with or without UV radiation. Therefore, the presence of this isoflavone does not alter cell proliferation, at least not during the first 48 h. Higher NDI values in the treated samples compared to the control samples were observed in 4′-L-Gen and WBn-Gen samples. Additionally, the higher the NDI value observed, the greater the number of MN cells present. Interestingly, the cells treated with a genistein derivative and subsequently exposed to UV radiation had much lower NDI values than the cells exposed to UV radiation. Therefore, the presence of isoflavones may minimize the cell division increased by UV exposure. Unfortunately, the number of cells with MN suggests that the investigated compounds cannot diminish DNA damage at the same time.

In the case of Me45 cells, NDI values in control samples were higher than in NHDFs (NDI_K,NHDF_ = ~1.3; NDI_K,Me45_ = ~1.7), which can be explained by the higher proliferation potential of cancer cells. Exposure to UV radiation did not affect NDI values significantly (K vs. K UV). However, importantly, Me45 cells do not divide as effectively after treatment with isoflavones. One possible explanation is that the presence of these substances may affect the cell cycle. All cells treated with the investigated compounds exhibited lower NDI values in comparison to control cells. Therefore, isoflavones may inhibit the growth of cancer cells.

### 2.5. Cell Cycle Analysis

**Figure 8:**
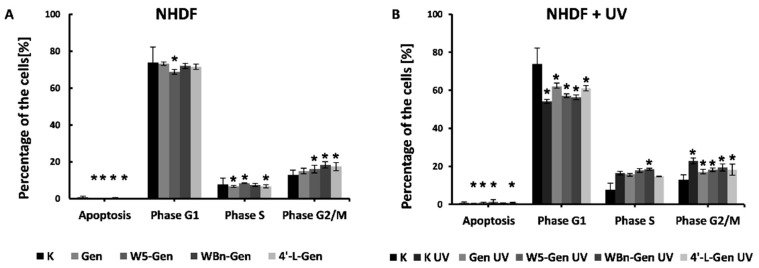
Effect of genistein and its derivatives on the cell cycle of NHDFs (**A**) and NHDFs exposed to UV light (**B**). Results are presented as mean ± SD calculated from three experiments and compared to the untreated controls (K). Statistical significance was calculated using the *t*-test where *p* < 0.05 and is indicated by an asterisk (*).

Based on the results of the MN assay, which suggests that the suppression of cell division is caused by the addition of isoflavones, the cell cycle of the cells treated with the investigated compounds was investigated more carefully. Figure 8 represents the percentage of NHDFs treated with genistein and its derivatives in various phases of the cell cycle, both not exposed (A) and exposed (B) to UV radiation.

A similar composition of the control and treated populations of cells (Figure 8A) indicates that the investigated compounds cause only a slight suppression of the cell cycle in the G2/M phase. The most significant suppression was observed for WBn-Gen-treated cells, which suppressed 6% of cells compared to the controls.

The increased number of cells in the G2/M (+10%) and S (+8%) phases in the K UV samples compared to the control samples (Figure 8B) indicates that UV radiation causes cell cycle suppression in these phases. A similar percentage of cells in the G2/M and S phases treated with various isoflavone compounds suggests that the investigated compounds do not affect the cell cycle, which agrees with the conclusions reached based on the NDI values.

**Figure 9:**
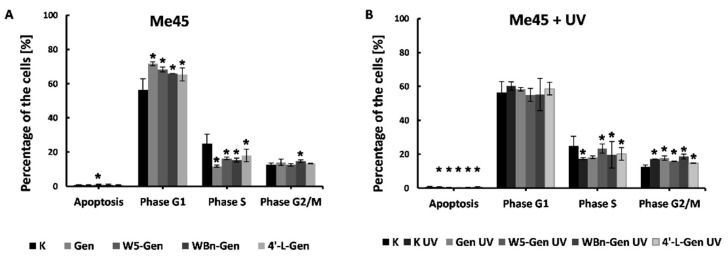
Effect of genistein and its derivatives on the cell cycle of Me45 cells (**A**), and those cells exposed to UV light (**B**). Results are presented as mean ±SD calculated from three experiments and compared to the untreated controls (K). Statistical significance was calculated using the *t*-test where *p* < 0.05 and is indicated by an asterisk (*).

Different observations were made in the Me45 cell line (Figure 9A). A much higher number of cells in the G1 phase (around 10–15%, depending on the compound), suggests that the addition of isoflavone compounds affects the cell cycle, causing suppression of the G1 phase and cell division. The strongest effect was caused by genistein, whereas the weakest effect was caused by treatment with 4′-L-Gen. Cell suppression in the G1 phase can be explained by insufficient amounts of nutrients or by the presence of agents that might cause DNA damage. In this case, the investigated compounds can be considered a harmful agent. The conclusion that genistein and its derivatives have a negative effect on Me45 cells agrees with the results of the MN assay, which identified increased DNA damage in the cells treated with the isoflavone compounds in comparison to the control cells.

Exposing Me45 cells to UV radiation (Figure 9B) changes the suppression of the G1 phase caused by the investigated compounds. The difference in the percentage of cells in the G1 phase in both the control and UV-irradiated control samples is ~5%. A similar difference is observed between the cells in the G2/S phase. Furthermore, there are no significant differences between the cells treated with the investigated compounds and the cells exposed to UV radiation, which can be explained by the effects of UV radiation on the cell cycle.

## 3. Materials and Methods

### 3.1. Reagents

Cytochalasin B (Cat. No. C2743); 2′,7′-dichlorodihydrofluorescein diacetate (H_2_DCFDA; Cat. No. D6883); dimethyl sulfoxide (DMSO; Cat. No. D8418); propidium iodide (PI; Cat. No. 537060); and ribonuclease (Cat. No. R6513) were purchased from Sigma Aldrich Co. (Schnelldorf, Germany). Methanol (Cat. No. 124790010); anhydrous ethanol (Cat. No. 397690010); ethylenediaminetetraacetic acid (EDTA; Cat. No. J62948.A1); and acetic acid (Cat. No. 148930010) were purchased from VWR (Radnor, PA, USA). Gentamycin (Cat. No. QJ01GB03) was purchased from Krka (Novo Mesto, Slovenia). Dulbecco’s modified Eagle’s medium (DMEM; Cat. No. P04-41250) from PanBiotech, Aidenbach, Germany), fetal bovine serum (FBS; Cat. No. E5050 from EUrX, Gdansk, Polska), Dulbecco’s phosphate buffered saline (PBS; Cat. No. P04-53500, PanBiotech, Aidenbach, Germany), and trypsin were purchased from PanBiotech (Cat. No. P10-0231SP, Aidenbach, Germany). 4′,6-Diamidino-2-phenylindole dihydrochloride (DAPI; Cat. No. D1306) was purchased from Thermo Fisher Scientific (Eindhoven, The Netherlands); 3-(4,5-dimethylthiazol-2-yl)-5-(3-carboxymethoxyphenyl)-2-(4-sulfophenyl)-2H-tetrazolium (MTS); and phenazine methosulfate (PMS) were purchased from Promega (Cat. No. G5421, Madison, WI, USA).

### 3.2. Genistein Derivatives

Genistein was obtained from the Pharmaceutical Institute in Warsaw, Poland. All tested genistein derivatives were synthesized by the Wiesław Szeja group (Department of Organic and Bioorganic Chemistry and Biotechnology, Silesian University of Technology, Gliwice, Poland). The investigated compounds are listed in Table 2.

High-purity genistein (>99% by HPLC) was used to synthesize the tested derivatives. The 7-*O*-alkyl derivatives of genistein were obtained by reacting the tetra-n-butylammonium salt of genistein (1 mmol) with an alkylating agent (1.1 mmol). However, the 4-*O*-alkyl derivative was obtained by reacting genistein (1.85 mmol) with an alkylating agent (1.85 mmol) in the presence of a strong base (6.10 mmol). The reactions were carried out in dry dimethylformamide (DMF) [46,47]. Tetra-n-butylammonium salt was obtained according to the procedure described in the literature [48]. HPLC analysis of the genistein derivatives was performed using a Dionex UHPLC-MS/MS system (Dionex Corporation, Sunnyvale, CA, USA) equipped with an UltiMate 3000 rapid separation (RS) pump, UltiMate 3000 spectrophotometric (UV-vis) detector, UltiMate 3000 thermostat, and UltiMate 3000 automatic sample feeder.

The UHPLC system was coupled to an API 4000 Q TRAP tandem mass spectrometer (Applied Biosystems/MDS SCIEX, Foster City, CA, USA). Analyses were performed at room temperature using a C18 ACE column (150 × 4.6 mm, 3.0 μm, Advanced Chromatography Technologies, UK). For the mobile phase, 15% of 0.1% solution formic acid in water (*v*/*v*) and 85% acetonitrile were used. [42]. The purity of the derivatives tested was ≥95%.

### 3.3. Cell Cultures

NHDFs were purchased from Lonza Group Ltd.—Lonza Verviers (Verviers, Belgium), and the human malignant melanoma (Me45) cell line was obtained from the Maria Skłodowska-Curie Memorial Cancer Center and National Institute of Oncology Branch Gliwice, Poland. Cells were cultured at 37 °C in 5% CO_2_ in DMEM-F12 medium supplemented with 12% FBS and 1% gentamycin using a Hera Cell incubator (Thermo Fisher Scientific, Eindhoven, The Netherlands). During the culturing periods, cells were passaged before confluency reached 80% in culture flasks and washed regularly with PBS. Before each test, cells were harvested by trypsinization (at a working concentration of 0.025%, prepared in a balanced salt solution without calcium or magnesium at a pH between 7.6–7.8), and neutralized with the same amount of DMEM. DMEM supplemented with 12% FBS and 1% gentamycin was used as the test medium in all of the tests.

### 3.4. Determination of EC_50_ Values—MTS Assay

The EC_50_ values of genistein and its derivatives for both cell lines were determined using the MTS assay (Cell Titer 96 AQueous One Solution Cell Proliferation Assay, Promega, Madison, WI, USA). Cells were seeded in 96-well plates (5 × 10^3^ cells/well) and incubated for 24 h at 37 °C in 5% CO_2_. Subsequently, the cells were treated with twelve concentrations of each compound ranging from 1–100 µM for 48 h at 37 °C in 5% CO_2_. Afterwards, the medium, including the investigated compounds, was removed from all wells and replaced with PBS. In total, 20 µL of MTS was added to each well. Plates were then incubated for 2 h at 37 °C in 5% CO_2_. The absorbance was measured at λ = 490 nm using an Epoch microplate reader (BioTek, Winooski, VT, USA). EC_50_ values were calculated using Matlab software (Matlab R2022a, MathWorks, Natick, MA, USA). The regression curves were fitted to data points using the following four-parametric Hill Equation (1):

(1) $$y=\min+\frac{max-min}{1+{\left(\frac{x}{{EC}_{50}}\right)}^{-HS}}$$

where y stands for the lethal effect [%], x is the compound concentration [µM], min and max are the minimal and maximal data values set to 0 and 100 [%], respectively, EC_50_ is the concentration causing 50% toxicity [µM], and HS is the Hill slope.

### 3.5. Test for Protective Potential against UV Irradiation—MTS Assay

Cells from each cell line were seeded in three groups (0, 24, and 48 h) in 96-well plates (5 × 10^3^ cells/well) and incubated for 24 h at 37 °C in 5% CO_2_. Afterwards, the medium in all wells was removed, and cells were treated with 5 µM of genistein, 4′-L-Gen, W-Bn-Gen, or W5-Gen. The chemicals added to the 0 h time point plates were dissolved in PBS. DMEM was used to prepare the chemical solutions for the 24- and 48-h plates.

After incubation, half of the wells on each plate were irradiated with UVC 2 kJ/m^2^ in a Crosslinker CL-1000 UV (UVP) device (Ultra-Violet Products Ltd., Cambridge, UK). The MTS assay was performed immediately after irradiation for 0 h plates and after 24 and 48 h of incubation for the other two time points. Before conducting the MTS test, the medium in the 24 and 48 h plates was discarded and replaced with PBS.

### 3.6. Quantification of Intracellular ROS

DMEM suspensions of both cell lines were placed in three groups (0, 24, and 48 h) of 12-well plates (2.5 × 10^4^ cells/well) and incubated for 24 h at 37 °C in 5% CO_2_. Medium in each well was replaced with fresh DMEM, and the cells were treated with 5 μM of genistein, 4′-L-Gen, W5-Gen, or WBn-Gen for 1 h at 37 °C in 5% CO_2_. Subsequently, half of the plates were irradiated according to the procedure described above. Cells from the 0 h time point plates were trypsinized, neutralized with DMEM, placed in Eppendorf tubes, and centrifugated at 2000 rpm for 3 min. The supernatant was removed, and the cells were suspended in 300 µL of DMEM with 30 µM H_2_DCFDA (dissolved in DMSO). Tubes were agitated using a vortex mixer and incubated with open lids for 30 min at 37 °C in 5% CO_2_. Next, the tubes were centrifugated at 2000 rpm for 3 min, and the supernatant was replaced with 300 µL of 4 °C PBS. Then, the tubes were agitated using a vortex mixer, stored on ice in the dark for 15 min, and agitated again before taking the measurements. Flow cytometry analysis was performed using the BD FACSCanto flow cytometer (Becton Dickinson, Franklin Lakes, NJ, USA). The first 5000 cells from each tube were considered during the fluorescence analysis.

### 3.7. Genotoxicity—Micronucleus Assay

Both cell types were seeded in 6-well plates (2.5 × 10^4^ cells/well) and incubated for 24 h at 37 °C in 5% CO_2_. After incubation, the medium in the wells was changed to 5 µM solutions of genistein, 4′-L-Gen, W-Bn-Gen, or W5-Gen in DMEM and incubated for 1 h at 37 °C in 5% CO_2_. Half of the treated cells were irradiated, as described above. Afterwards, all the cells were treated with 1 mg/mL cytochalasin B and incubated for 48 h at 37 °C in 5% CO_2_. Subsequently, cells from each well were trypsinized, washed twice in PBS, and mixed with frosted (−20 °C) Carnoy’s solution (methanol: acetic acid, *v*/*v* 3:1). After 2 h, 50 µL of each suspension was placed on a microscopic slide and dried. Before microscopic analysis, slides were stained with 2 µg/mL DAPI (dissolved in DMSO) and covered with cover glasses. Slides were observed using a fluorescent microscope (Zeiss Axio Imager. M1 with EC Plan- NEOFLUAR 40× objective, Zeiss, Oberkochen, Germany). A total of 500 cells on each slide were counted, taking into account binucleated, binucleated with MN, mononucleated, and apoptotic cells. The NDI was calculated using a modified method by Eastmond and Tucker (1989) according to the following Equation (2) [49]:

(2) $$NDI=\frac{MNu+2(Bnu+{BNu}_{MN})}{\sum cells}$$

where MNu is the number of cells with one nucleus, BNu is the number of binucleated cells, and BNuMN is the number of binucleated cells with MN.

### 3.8. Cell Cycle Analysis

Cells from both cell lines were seeded in 12-well plates (2.5 × 10^4^ cells/well) and incubated for 24 h at 37 °C in 5% CO_2_. The medium in the wells was replaced with fresh DMEM. Cells were treated with 5 µM of genistein, 4′-L-Gen, W5-Gen, or WBn-Gen for 48 h. Medium containing the test substances was removed, and the cells were collected by trypsinization (followed by neutralization of trypsin with medium) in Eppendorf tubes. Tubes were centrifuged (2000 rpm, 3 min), and the supernatant was removed. Subsequently, the fixation of cells in frozen 96% ethanol took place. The ethanol was removed, and cells were washed twice in PBS. Cells were suspended in tubes using 50 µL of 100 µg/L RNase and then incubated for 15 min at room temperature. Cells were stained with 100 µg/L propidium iodide solution in PBS and incubated for 20 min at room temperature. Directly before flow cytometry analysis, cells were agitated on a vortex mixer. Cell cycle analysis was performed using a BD FACS Canto flow cytometer, which counted 10,000 cells from every probe. The number of cells in each cell cycle phase was counted by measuring the luminescence intensity using Diva software (Bd FACSDiva v9.0, Becton Dickinson, Franklin Lakes, NJ, USA).

### 3.9. Statistical Analysis

All experiments were repeated three times, and the data were shown as mean ± standard error (SE) of three assays. Student’s *t*-test was applied, and *p* < 0.05 was considered statistically significant.

## 4. Conclusions

This study proved that the modification of genistein’s structure affects its biological activity. The addition of a lipophilic functional group increases the interaction with cell membranes and may increase the protective effect on cells, but it is highly dependent on the cell type.

The calculated EC_50_ values indicate that all of the investigated genistein derivatives are more toxic for NHDFs than genistein, but less toxic for Me45 cells (except for W5-Gen). There is no clear dependence between the effect of lipophilicity, expressed as log P, and the biological activity, which indicates that the structure of the lipophilic part likely plays an important role in the biological effect on cells.

Detailed monitoring of the cell viability for cells treated with the investigated isoflavones suggested that the investigated compounds exhibit much higher protective potential towards cancer cells than healthy cells. However, the investigated compounds can also protect healthy cells from the negative effects of UV radiation, especially at 48 h post-exposure to UV.

Quantification of intracellular ROS proved that the protective characteristics of genistein and its derivatives against UVC irradiation in NHDFs are negligible compared to Me45 cells. The compounds would probably not protect healthy cells from irradiation’s toxic effects but possibly promote cancer progression, which is neither an expected nor a desired effect.

The investigated compounds can also be genotoxic. Me45 cells seemed to be more susceptible to DNA damage than NHDFs. Only the W5-Gen derivative exhibited a slight protective effect against UV radiation exposure.

We believe that our study significantly contributes to a better understanding of how the modification of compound structures can affect the biological activity. Moreover, we hope that our results can be useful for designing new genistein derivatives with improved lipophilicity as well as better antioxidative properties.

## Figures and Tables

**Table 1:** EC_50_ values [µM] of the investigated compounds in NHDFs and Me45 cells.

	NHDF			Me45		
Compound	EC_50_ (±SE *)	R^2^	Hill Slope	EC_50_ (±SE *)	R^2^	Hill Slope
Gen	8.6 (±1.6)	0.9084	0.7206	78.2 (±9.9)	0.8526	0.9270
W5-Gen	5.6 (±1.2)	0.9000	0.5870	92.2 (±6.9)	0.7767	3.1178
WBn-Gen	6.0 (±1.7)	0.8324	0.5911	71.2 (±10.1)	0.6856	2.0406
4′-L-Gen	6.5 (±2.4)	0.7267	0.5275	60.5 (±5.8)	0.8741	3.0014

* SE—standard error.

**Table 2:** Genistein and its derivatives investigated in the study.

Abbreviated Name	Compound Name	Structural Formula	Molar Mass	Log P
Gen	Genistein	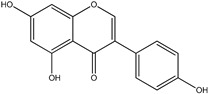	270.24	1.74
W5-Gen	n-pentyl-7-*O*- genistein carbonate	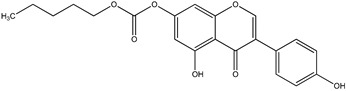	384.34	3.96
WBn-Gen	bezyl-7-*O*- genistein carbonate	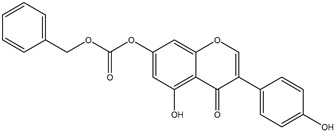	404.37	4.04
4′-L-Gen	4′-genistein dodecanate (Common name genistein laurate)	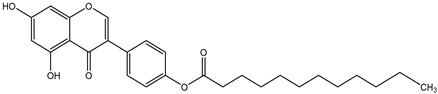	452.54	5.71

## Data Availability

The raw data supporting the conclusions of this article will be made available by the authors upon request.

## Supplementary Materials

The following supporting information can be downloaded at: https://www.mdpi.com/article/10.3390/ph17091166/s1, Table S1: statistics for Figure 4, Figure 5, Figure 6, Figure 7, Figure 8 and Figure 9. Figure S1: representative cell cycle histograms of control NHDF and Me45 cells (A); after compounds addition (B); and compounds with UV radiation (C). Figure S2: representative ROS level histograms of control NHDF and Me45 cells (A); after compounds addition (B); and compounds with UV radiation (C). Figure S3: representative images of nucleus of binucleated control NHDF and Me45 cells (A); after compounds addition (B); and compounds with UV radiation (C). Fluorescence microscopy images after DAPI staining; magnification 100× (A,B) or 400× (C). Figure S4: viability of cells presented as survival fraction, SF [%], in comparison to the untreated controls. The results are presented as the mean from at least three experimental repeats, +/− SD (5%). Table S2: statistics for Figure S4.
