# Supplementary material for: In Vitro Studies of Genistein Lipophilic Derivatives as Potential UV Radiation Protectors
Source: Pharmaceuticals (Basel). 2024 Sep 3;17(9):1166. doi: 10.3390/ph17091166 (PMC11435217; doi:10.3390/ph17091166)
Supplement: Supplementary file 1 [file pharmaceuticals-17-01166-s001.zip › pharmaceuticals-3065751-supplementary.pdf]

Supplementary files

Results for **Figures 4-9** are presented as mean, ± SD calculated from 3 experiments and compared to the untreated controls (K) - at 0 h or 1 h time point, if timeframe is indicated, respectively. Statistical significance was calculated from a *t*-test where $p < 0.05$ and indicated by an asterisk (*). Below in Tables are bolded.

Table S1. Statistics for Figures 4-9

Figure 4

A

NHDF					Me45				
	k	k+UV	Gen	Gen+UV		k	k+UV	Gen	Gen+UV
0h	0.5	**0.00159**	0.13878	**0.00014**	0h	0.5	**0.00812**	**7.13E-05**	**3.32E-05**
24h	**0.00019**	**2.12E-05**	**3.22E-05**	**0.00019**	24h	**0.00059**	**0.00086**	0.5	0.07576
48h	**0.00283**	**1.45E-05**	**0.00033**	**0.00052**	48h	**0.00033**	0.29764	**0.00175**	**0.00041**

B

NHDF					Me45				
	k	k+UV	W5-Gen	W5-Gen+UV		k	k+UV	W5-Gen	W5-Gen+UV
0h	0.5	**0.00159**	**0.00283**	**0.00834**	0h	0.5	**0.00812**	**7.13E-05**	**3.32E-05**
24h	**0.00019**	**2.12E-05**	**8.32E-05**	**1.38E-05**	24h	**0.00059**	**0.00086**	0.5	0.07576
48h	**0.00283**	**1.45E-05**	**0.00283**	**0.00114**	48h	**0.00033**	0.29764	**0.00175**	**0.00041**

C

NHDF					Me45				
	k	k+UV	WBn-Gen	WBn-Gen+UV		k	k+UV	WBn-Gen	WBn-Gen+UV
0h	0.5	**0.00159**	**0.00834**	**0.00114**	0h	0.5	**0.00812**	**3.68E-05**	**4.08E-05**
24h	**0.00019**	**2.12E-05**	**0.00114**	**5.07E-05**	24h	**0.00059**	**0.00126**	0.39437	0.05322
48h	**0.00283**	**1.45E-05**	**0.00098**	0.13878	48h	**0.00033**	0.29764	**0.00017**	**0.00073**

D

NHDF					Me45				
	k	k+UV	4′-L-Gen	4′-L-Gen+UV		k	k+UV	4′-L-Gen	4′-L-Gen+UV
0h	0.5	**0.00159**	**0.00065**	**0.00834**	0h	0.5	**0.00375**	**0.00812**	**0.00036**
24h	**0.00019**	**2.12E-05**	**0.04012**	**3.22E-05**	24h	**0.00043**	**0.00091**	0.37456	0.09853
48h	**0.00283**	**1.45E-05**	**0.00033**	**0.00283**	48h	**0.00033**	0.29764	0.05322	**0.00621**

Figure 5

A

NHDF					Me45				
	k	k+UV	Gen	Gen+UV		k	k+UV	Gen	Gen+UV
1h	0.5	**0.00423**	**0.00198**	**0.01648**	1h	0.5	**0.01352**	**0.01063**	**0.00159**
24h	0.5	0.08656	**0.00283**	0.13878	24h	0.5	**0.00143**	**0.02521**	**0.00061**
48h	0.5	**0.00569**	**0.00423**	**0.00489**	48h	0.5	0.40977	**0.01352**	0.18704

B

NHDF					Me45				
	k	k+UV	Gen	Gen+UV		k	k+UV	W5-Gen	W5-Gen+UV
1h	0.5	**0.00423**	**0.00569**	**0.00098**	1h	0.5	**0.01352**	0.14913	**0.00016**
24h	0.5	**0.08656**	**0.00346**	**0.00661**	24h	0.5	**0.00143**	0.40972	**0.00283**
48h	0.5	**0.00177**	**0.02328**	**0.00423**	48h	0.5	0.40972	0.05138	**0.19562**

C

NHDF					Me45				
	k	k+UV	Gen	Gen+UV		k	k+UV	W5-Gen	W5-Gen+UV
1h	0.5	**0.00423**	**0.00143**	**0.00368**	1h	0.5	**0.01352**	**0.03083**	**0.00423**
24h	0.5	0.08656	0.13878	**0.03164**	24h	0.5	**0.00143**	**0.00021**	**0.00008**
48h	0.5	**0.00177**	0.32651	**0.00368**	48h	0.5	0.40972	0.5	0.32651

D

NHDF					Me45				
	k	k+UV	Gen	Gen+UV		k	k+UV	W5-Gen	W5-Gen+UV
1h	0.5	**0.00423**	0.11347	**0.01648**	1h	0.5	**0.01352**	0.40972	**0.00159**
24h	0.5	0.08656	**0.00024**	**0.01063**	24h	0.5	**0.00143**	**0.00011**	**0.00233**
48h	0.5	**0.00177**	**0.00039**	0.5	48h	0.5	0.40972	**0.00014**	0.19562

Figure 6

A

NHDF

k	k+UV	Gen	Gen+UV	W5-Gen	W5-Gen+UV	WBn-Gen	WBn-Gen+UV	4'-L-Gen	4'-L-Gen+UV
0.5	**0.00001**	**0.00007**	**0.00005**	**4.32E-06**	**0.00001**	**5.96E-06**	**5.23E-06**	**0.00001**	**4.91E-06**

B

Me45

k	k+UV	Gen	Gen+UV	W5-Gen	W5-Gen+UV	WBn-Gen	WBn-Gen+UV	4-L-Gen	4-L-Gen+UV
0.5	**0.00011**	**0.00001**	**0.00002**	**0.000065**	**0.00329**	**0.00006**	**0.00005**	**0.00001**	**0.00002**

Figure 7

A

NHDF

k	k+UV	Gen	Gen+UV	W5-Gen	W5-Gen+UV	WBn-Gen	WBn-Gen+UV	4-L-Gen	4-L-Gen+UV
0.5	**0.00376**	0.5	0.35862	0.42851	**0.02211**	**0.00174**	0.19895	**0.00471**	**0.16078**

B

Me45

k	k+UV	Gen	Gen+UV	W5-Gen	W5-Gen+UV	WBn-Gen	WBn-Gen+UV	4-L-Gen	4-L-Gen+UV
0.5	0.22772	**0.00077**	**0.00283**	**0.011411**	**0.0045238**	**0.001225**	**0.00452**	**0.00065**	**0.00356**

Figure 8

A

NHDF

	k	Gen	W5-Gen	WBn-Gen	4-L-Gen
apoptosis	0.5	**0.00001**	**0.00002**	**0.00003**	**0.00001**
G0/G1	0.5	0.41958	**0.07742**	0.29426	0.24539
S	0.5	**0.01401**	**0.06008**	0.19422	**0.01189**
G2/M	0.5	0.49331	**0.00316**	**0.00054**	**0.00102**

B

NHDF+UV

	k	k+UV	Gen+UV	W5-Gen+UV	WBn-Gen+UV	4-L-Gen+UV
apoptosis	0.5	**0.00001**	**0.00193**	**0.00007**	0.19562	**0.00435**
G0/G1	0.5	**0.00088**	**0.00685**	**0.00175**	**0.00142**	**0.00506**
S	0.5	0.22441	0.15019	0.09342	0.24369	0.35339
G2/M	0.5	**0.00009**	**0.00131**	**0.00061**	**0.00032**	**0.00059**

Figure 9

A

Me45

	k	Gen	W5-Gen	WBn-Gen	4-L-Gen
apoptosis	0.5	0.19562	**0.00435**	0.05171	0.28449
G0/G1	0.5	**0.00217**	**0.00472**	**0.00953**	**0.01138**
S	0.5	**2.26E-06**	**0.00028**	**0.00017**	**0.00072**
G2/M	0.5	0.20228	0.47808	**0.00835**	0.12237

B

Me45+UV

	k	k+UV	Gen+UV	W5-Gen+UV	WBn-Gen+UV	4-L-Gen+UV
apoptosis	0.5	**0.00159**	**0.00001**	**0.00001**	**0.00001**	**0.00159**
G0/G1	0.5	0.08978	0.22383	0.29507	0.31588	0.18967
S	0.5	**0.00079**	0.08498	**0.02862**	**0.00321**	**0.00343**
G2/M	0.5	**0.00081**	**0.00059**	**0.00249**	**0.00036**	**0.00779**

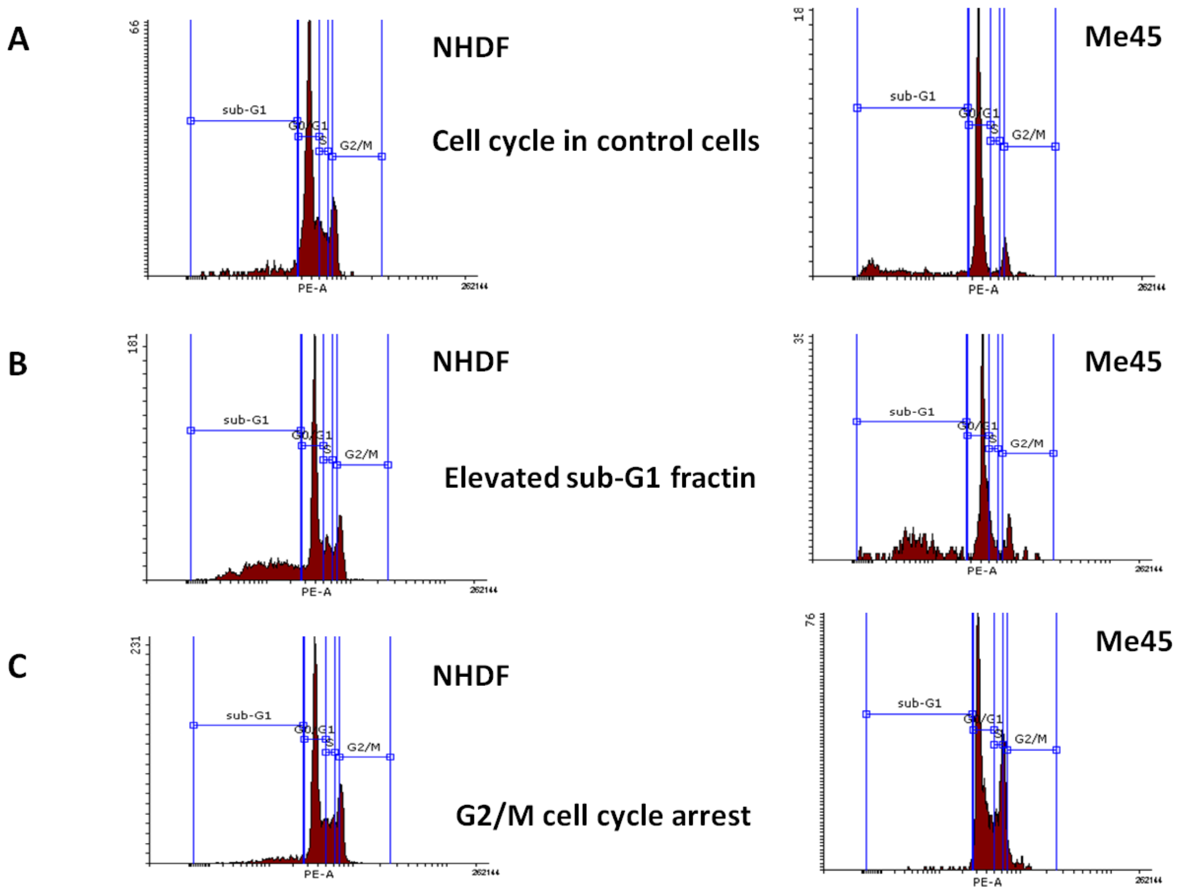

Figure S1. Representative cell cycle histograms of control NHDF and Me45 cells (A); after compounds addition (B) and compounds with UV radiation (C).

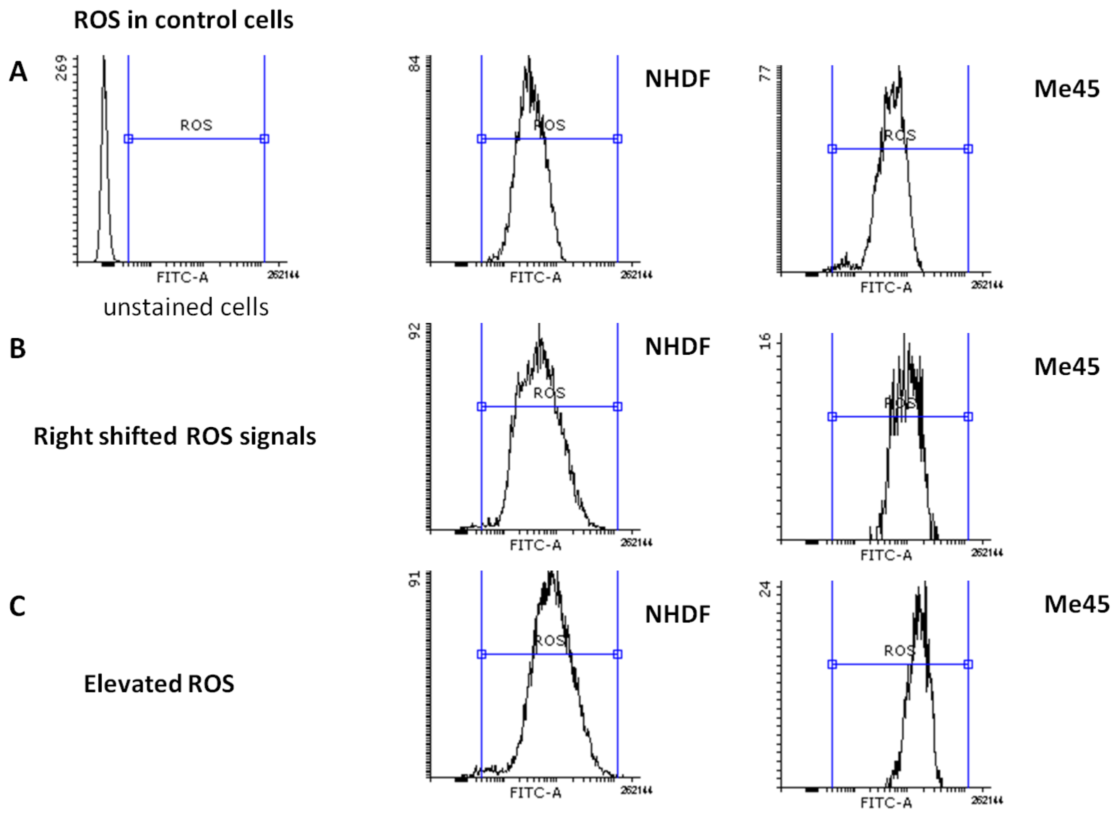

Figure S2. Representative ROS level histograms of control NHDF and Me45 cells (A); after compounds addition (B) and compounds with UV radiation (C).

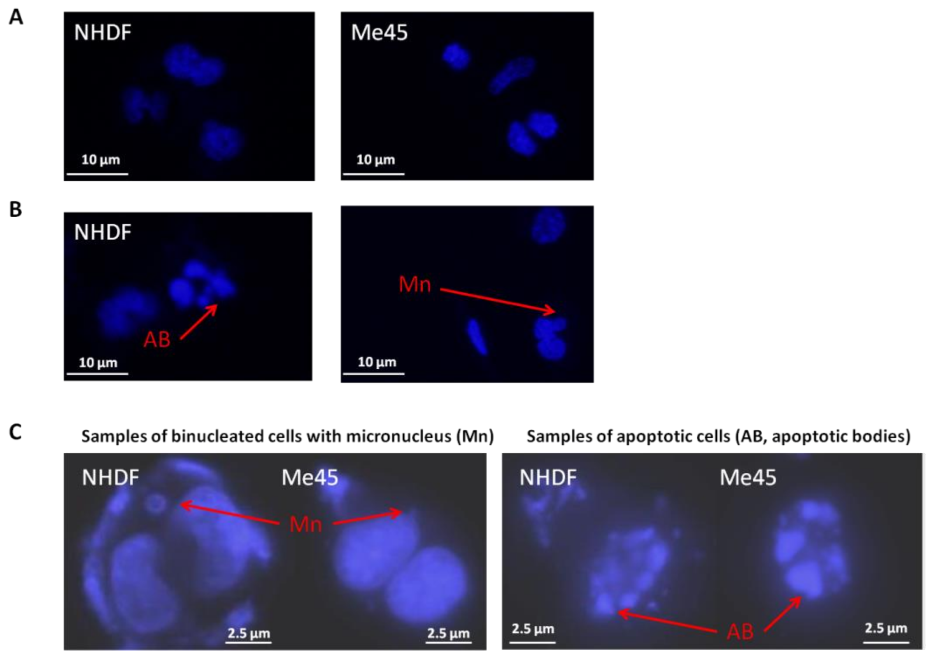

Figure S3. Representative images of nucleus of binucleated control NHDF and Me45 cells (A); after compounds addition (B) and compounds with UV radiation (C). Fluorescence microscopy images after DAPI staining; magnification 100x (A and B) or 400x (C).

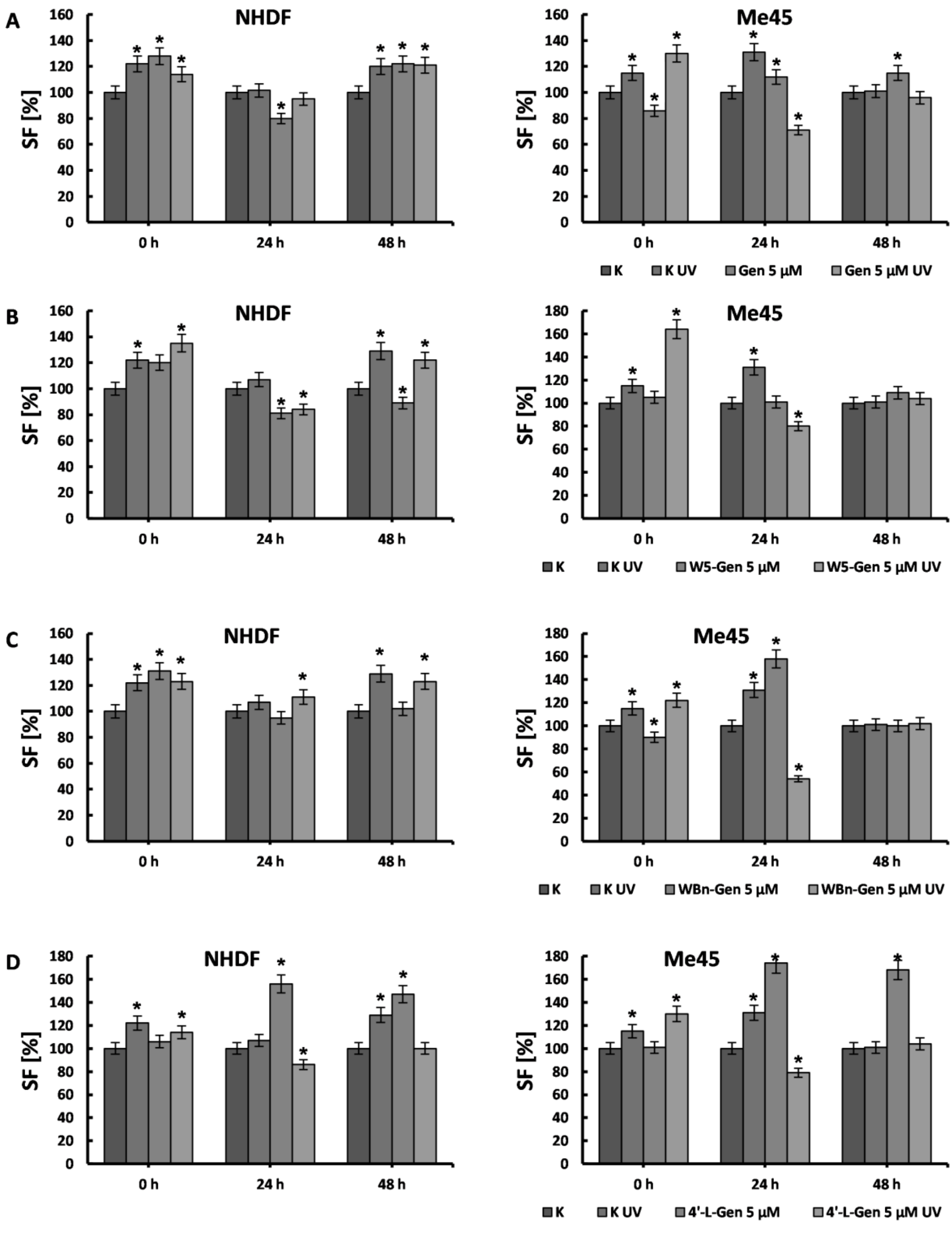

Figure S4. Viability of cells presented as Survival Fraction, SF [%], in comparison to the untreated controls. The results presented as mean from at least 3 experimental repeats, +/- SD (5%).

Statistical significance was calculated from a *t*-test where $p < 0.05$ and indicated by an asterisk (*). Below in Tables are bolded.

Table S2. Statistics for Figure S4

A

NHDF					Me45				
k	k	k+UV	Gen	Gen+UV	k	k	k+UV	Gen	Gen+UV
0h	0.5	**0.00423**	**0.00198**	**0.01648**	0h	0.5	**0.01352**	**0.01063**	**0.00159**
24h	0.5	0.08656	**0.00283**	0.13878	24h	0.5	**0.00143**	**0.02521**	0.00061
48h	0.5	**0.00569**	**0.00423**	**0.00489**	48h	0.5	0.40972	**0.01352**	0.18704

B

NHDF					Me45				
k	k	k+UV	Gen	Gen+UV	k	k	k+UV	W5-Gen	W5-Gen+UV
0h	0.5	**0.00423**	**0.00569**	**0.00098**	0h	0.5	**0.01352**	0.14913	**0.00016**
24h	0.5	0.08656	**0.00345**	**0.00661**	24h	0.5	**0.00143**	0.40972	**0.00283**
48h	0.5	**0.00178**	**0.02328**	**0.00423**	48h	0.5	0.40972	0.05138	0.19562

C

NHDF					Me45				
k	k	k+UV	Gen	Gen+UV	k	k	k+UV	W5-Gen	W5-Gen+UV
0h	0.5	**0.00423**	**0.00143**	**0,003676841**	0h	0.5	**0.01352**	**0.03083**	**0.00423**
24h	0.5	0.08656	0.13878	**0,031635701**	24h	0.5	**0.00143**	**0.00021**	**7.51E-05**
48h	0.5	**0.00177**	0.32651	**0,003676841**	48h	0.5	0.40972	0.5	0.32651

D

NHDF					Me45				
	k	k+UV	Gen	Gen+UV		k	k+UV	W5-Gen	W5-Gen+UV
0h	0.5	**0.00423**	0.11347	**0.01648**	0h	0.5	**0.01352**	0.40972	**0.00159**
24h	0.5	0.08656	**0.00024**	**0.01063**	24h	0.5	**0.00143**	**0.00011**	**0.00233**
48h	0.5	**0.00177**	**0.00039**	0.5	48h	0.5	0.40972	**0.00014**	**0.19562**
